# Genomic Analysis of KPC-2-Producing *Klebsiella pneumoniae* ST11 Isolates at the Respiratory Department of a Tertiary Care Hospital in Beijing, China

**DOI:** 10.3389/fmicb.2022.929826

**Published:** 2022-06-16

**Authors:** Ling Guo, Lifeng Wang, Qiang Zhao, Liyan Ye, Kun Ye, Yanning Ma, Dingxia Shen, Jiyong Yang

**Affiliations:** Department of Laboratory Medicine, First Medical Center of Chinese PLA General Hospital, Beijing, China

**Keywords:** genomic, *Klebsiella pneumoniae*, ST11, KPC-2, hypervirulence

## Abstract

**Background:**

Carbapenem-resistant *Klebsiella pneumoniae* (CRKP) is an important pathogen causing hospital-associated outbreaks worldwide. The spread of *K. pneumoniae* carbapenemase-2 (KPC-2)-producing CRKP is primarily associated with sequence type (ST) 11.

**Methods:**

A total of 152 KPC-2-producing *K. pneumoniae* ST11 isolates were collected from the respiratory department of a tertiary care hospital in Beijing, China between 2009 and 2018. The genome sequencing of these isolates was performed on the HiSeq X Ten sequencer. Multilocus sequence typing (MLST), capsular type, plasmid replicon types and resistance genes were identified. Fifteen isolates were selected for the subsequent single-molecule real-time (SMRT) sequencing on the PacBio RS II. Alignment of the complete sequences of the plasmids carrying *bla*_KPC–2_ and/or virulence genes was performed by using BRIG and Easyfig.

**Results:**

From 2012 to 2018, the detection rate of the *bla*_KPC–2_-carrying CRKP rose rapidly from 3.3 to 28.1%. KPC-2-producing *K. pneumoniae* ST11 isolates were dominant in CRKP, which emerged in 2012 and caused several outbreaks. Most isolates exhibited multidrug-resistant to commonly used antibiotics, while all the isolates remained susceptible to tigecycline and polymyxin B. The single nucleotide polymorphism (SNP) analysis showed that all these 152 KPC-2-producing *K. pneumoniae* ST11 isolates could be divided into three genetically distinct clades (A, B, and C) and eleven subclades (A1–A9 and B1–B2). The majority belonged to clade A with KL47 serotype (*n* = 117, 77.0%), while KL64 and KL16 were identified in clades B and C, respectively. The *bla*_KPC–2_-carrying plasmids exhibited diverse types, namely, IncFII (pHN7A8)/IncR(6/15), IncFII (pHN7A8)/Inc_pA1763–KPC_ (5/15), IncFII (pHN7A8) (1/15), IncR (1/15), and Inc_pA1763–KPC_ (1/15). The genetic environment of *bla*_KPC–2_ showed nine IS*26*-based composite transposons, which had a basic core structure IS*Kpn27-bla*_KPC–2_-ΔIS*Kpn6*. About 27.6% (42/152) isolates co-carried 2 to 4 virulence marker genes (namely, *peg344*, *iucA*, *iroB*, *rmpA*, and *rmpA2*) for hvKp strains. At least three isolates were identified to harbor virulence gene-carrying plasmids.

**Conclusion:**

KPC-2-producing *K. pneumoniae* ST11 was highly heterogeneous in our hospital. Transmission of these strains was mainly mediated by twelve high-risk clones. The *bla*_KPC–2_-carrying plasmids and genetic environment of *bla*_KPC–2_ genes exhibited active evolution in *K. pneumoniae* ST11. More attention should be paid to the tendency of KPC-2-ST11 to acquire hypervirulent plasmids.

## Introduction

The increasing prevalence of carbapenem-resistant *Klebsiella pneumoniae* (CRKP) poses a continuously increasing threat to public health worldwide (Munoz-Price et al., 2013). The production of carbapenemases, namely, *K. pneumoniae* carbapenemase (KPC), NDM, and OXA-48, is the most important mechanism among carbapenem-resistant *K. pneumoniae* (Lee et al., 2016). Among these carbapenemases, KPC is the most prevalent enzyme since it was reported in 1996 (Nordmann and Poirel, 2019). In China, about 60% of CRKP strains produce the KPC and the KPC-2 is the major variant of KPC family (Hu et al., 2020). Transmission of the KPC-2-coding gene involves multiple mechanisms ranging from clonal spread to horizontal transfer mediated by plasmids and other transposons (Naas et al., 2008).

The spread of KPC-producing *K. pneumoniae* is primarily associated with different sequence types (STs). ST258 is most common in North America, Latin America, and Europe, while ST11 is most common in Asia. ST11 is a single-locus variant (*tonB*) of ST258 and both of them belong to clone complex CC258. In China, the mass dissemination of KPC-2-producing CRKP has been primarily restricted to clone ST11 (Yang et al., 2013; Cheng et al., 2016; Shen et al., 2016; Wang et al., 2016; Gu et al., 2019; Li et al., 2019). The genomic analysis has revealed that ST11 strains were highly heterogeneous and can be grouped into two or three genetic lineages according to the capsule types, while KL47 and KL64 are the main types, which have caused outbreaks in many districts (Dong et al., 2018b; Zhao et al., 2020).

The pandemic spread of *bla*_KPC–2_ among ST11 CRKP in China is mainly associated with horizontal transfer mediated by incompatibility group F (IncF) plasmids. The IncFII replicon often coexists with one or more other replicons such as IncFIA (Shen et al., 2016), IncFIB (Wang et al., 2016), and IncR (Yang et al., 2013). The IncpA1763-KPC replicon is a new type of replicon and was designated in 2019 (Qu et al., 2019). Plasmids with multireplicon have a better chance to replicate in a broader range of hosts by encoding different sets of replication initiation proteins (Li et al., 2019). There were also some new types of plasmids carrying *bla*_KPC–2_ discovered.

Genetic structures surrounding the *bla*_KPC–2_ gene are mostly associated with a Tn*3*-based transposon, Tn*4401*, with *bla*_KPC–2_ sandwiched between insertion sequences, namely, IS*Kpn6* and IS*Kpn7* (Naas et al., 2008). The genetic environments of *bla*_KPC–2_ from China can be assigned into three main categories: Tn*4401*, the Tn*1722*-based unit transposon Tn*6296*, and the IS*26*-based truncated Tn*6296* (ΔTn*6296*). Both the Tn*6296* and ΔTn*6296* contain the IS*Kpn27-bla*_KPC–2_-IS*Kpn6* core structure (Zhang et al., 2017). The ΔTn*6296* has one or two IS*26* elements at one or both ends, while different lengths of truncated Tn*6296* can be observed from different plasmids.

Due to the rapid evolution of ST11, carbapenem-resistant hypervirulent ST11 has also been consistently reported (Dong et al., 2018b; Zhao et al., 2020), which has led to a growing therapeutic dilemma (Chen et al., 2018; Gu et al., 2018).

In our hospital, a nosocomial outbreak of CRKP was first reported in 2013 and KPC-2-producing CRKP ST11 was the main pathogen of the transmission (Yang et al., 2013). To further characterize the nosocomial transmission and genomic characteristics of this high-risk clone, all the KPC-2-producing CRKP ST11 isolated from the respiratory department were collected from 2010 to 2018 and their genomic characteristics were analyzed.

## Materials and Methods

### Bacterial Strains

All the non-duplicated *K*. *pneumoniae* isolates were consecutively collected from various clinical specimens between 2010 and 2018 at the respiratory department, including four general wards and one intensive care unit (ICU) in our hospital. All the clinical isolates were identified using VITEK 2 GN cards or VITEK MS (bioMérieux SA, Marcy-l’Etoile, France). The isolates that exhibited non-susceptibility to carbapenems were screened by PCR amplification for the common carbapenemase genes, namely, *bla*_KPC_, *bla*_NDM_, *bla*_OXA–48_, and *bla*_IMP_, as described previously (Poirel et al., 2011). Isolates harboring *bla*_KPC_ genes were further analyzed.

### Antimicrobial Susceptibility Test

The susceptibility of strains was tested by the VITEK 2 or KB method at first. The minimal inhibition concentrations (MICs) of cefotaxime (CTX), ceftazidime (CAZ), piperacillin-tazobactam (TZP), imipenem (IMP), meropenem (MEM), ertapenem (ETP), amikacin (AK), ciprofloxacin (CIP), tigecycline (TGC), sulfamethoxazole/trimethoprim (SXT), and polymyxin B (PB) were measured with broth microdilution method by using the Biofosun^®^ Gram-negative panels (Biofosun Biotechnology Corporation Ltd., Shanghai, China) for 15 strains of complete genome sequencing. Results were interpreted according to the interpretive standards of the Clinical and Laboratory Standards Institute (M100, 30th edition). Susceptibility to tigecycline and polymyxin B was interpreted according to the 2017 European Committee on Antimicrobial Susceptibility Testing (EUCAST) breakpoints (Supplementary Table 1).

### Whole-Genome Sequencing

Genome DNA from overnight cultures was extracted using the DNeasy^®^ UltraClean^®^ Microbial Kit (QIAGEN GmbH, 40,724 Hilden, Germany). Genome sequencing was performed with a paired-end library with an average insert size of 350 bp on the HiSeq X Ten sequencer (Illumina, California, CA, United States). The draft genome was assembled into a scaffold. Quality assessment was performed with Fastqc (Version 0.11.8) and all reads score above Q30 were used with follow-up analysis. After removing the adapter, barcode, and trimming of the raw reads, sequences were assembled using SOAP *de novo* (SOAP version 2.21) with default settings. N50, N90, coverage rate, and scaffold number were used to identify *de novo* characters.

### Data Analysis

*Klebsiella* multilocus sequence typing^1^ databases were performed to identify ST using data from the assembled contigs. Whole-genome sequencing (WGS) data for all the isolates were analyzed using ResFinder and PlasmidFinder tools provided by the Center for Genomic Epidemiology^2^ to identify genes encoding antimicrobial resistance and plasmid replicons using an identity threshold of 95% and a selected minimum length of 60%. Determination of capsular type and *wzi* alleles was conducted by using Kleborate.^3^ The presence of the virulence genes or gene clusters was screened by using the reference databank of the Pasteur Institute, Kleborate, and the National Center for Biotechnology Information (NCBI) blast. We screened for genes associated with increased pathogenicity, namely, *peg344*, *iucA* (aerobactin siderophore gene), *iroB* (salmochelin siderophore biosynthesis gene), *rmpA*, and *rmpA2* (regulators of the mucoid phenotype *via* increased capsule production), which could be markers for hypervirulent strains and demonstrated >0.95% diagnostic accuracy for identifying hypervirulent *K. pneumoniae* (Russo et al., 2018; Supplementary Table 2).

### Phylogenic Analysis

The CSI Phylogeny version 1.4^4^ was used to call single nucleotide polymorphism (SNP) and alignment. The complete genome sequence of ST11 *K. pneumoniae* strain HS11286 (CP003200.1) was used as a reference. The tree file was visualized by iTOL^5^ and annotated information was edited by iTOL editor v1_1.

### Complete Genome Sequencing Analysis

One or two strains from each subclade were selected for subsequent single-molecule real-time (SMRT) sequencing using a sheared DNA library with an average size of 10 kb on the PacBio RS II (Pacific Biosciences, Menlo Park, CA, United States), and a paired-end library with an average insert size of 350 bp on an Illumina NovaSeq platform (Illumina, California, CA, United States). Preliminary assembly was performed using the Hierarchical Genome Assembly Process 3 (HGAP3) within SMRT link version 5.0.1. The paired-end short Illumina reads were used to correct the polymer errors for long PacBio reads. Reads were mapped into assembled genome sequences using BWA version 0.5.9. Pilon version 1.13 was subsequently used to polish genome sequences using the obtained alignments. The assemblies yielded a circular chromosome and several plasmids for each strain. Genome sequences were annotated with the Rapid Annotation using Subsystem Technology (RAST) pipeline (Overbeek et al., 2014), screening for insertion sequences in ISfinder (Siguier et al., 2006) and BLAST searches. Alignment of *bla*_KPC–2_-carrying plasmid complete sequences of each strain used BRIG and Easyfig.

### GenBank Accession Number

All the genome sequences used in this study were submitted to GenBank with BioProject accession numbers PRJNA782392 and PRJNA835045.

## Results

### Clinical Characteristics

A total of 1,043 non-duplicated *K. pneumoniae* isolates were collected from various clinical specimens of the respiratory departments between 2010 and 2018, of which about 20.6% (*n* = 215) were carbapenem-insensitive CRKPs. The majority of CRKP carried *bla*_KPC–2_ gene (*n* = 170, 79.1%), *bla*_OXA–48_ (*n* = 39, 18.1%), and *bla*_IMP_ (*n* = 3, 1.4%). All the 173 *bla*_KPC–2_-carrying strains were isolated from various clinical sources from 170 unique patients. Almost half (49.7%) of CRKP was isolated from the respiratory intensive care unit (RICU). The most common sources were sputum (74.6%), urine (14.5%), and blood (5.2%). From 2012 to 2018, the detection rate of the *bla*_KPC–2_-carrying CRKP rose rapidly from 3.3 to 28.1%. Most isolates exhibited multidrug-resistant to commonly used antibiotics, while all the isolates remained susceptible to tigecycline and polymyxin B (Supplementary Table 1).

### Multilocus Sequence Typing Analysis

**FIGURE 1:**
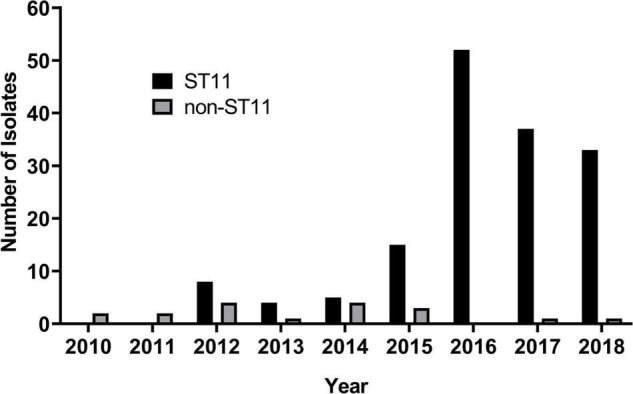
The number of *Klebsiella pneumoniae* carbapenemase-2 (KPC-2) producing carbapenem-resistant *K. pneumoniae* (CRKP) strains isolated each year.

Among the 170 *bla*_KPC–2_-carrying CRKP, six STs were identified. Approximately, 89.4% (*n* = 152) of them belonged to ST11, followed by ST15 (*n* = 9), ST395 (*n* = 5), ST392 (*n* = 2), ST163 (*n* = 1), and ST13 (*n* = 1). CRKP ST11 was first discovered in January 2012 and quickly became the main epidemic clone in our hospital (Figure 1).

### Core-Genome Phylogenetic Analyses of *Klebsiella pneumoniae* Carbapenemase-2 Producing Carbapenem-resistant *Klebsiella Pneumoniae* Sequence Type 11

**FIGURE 2:**
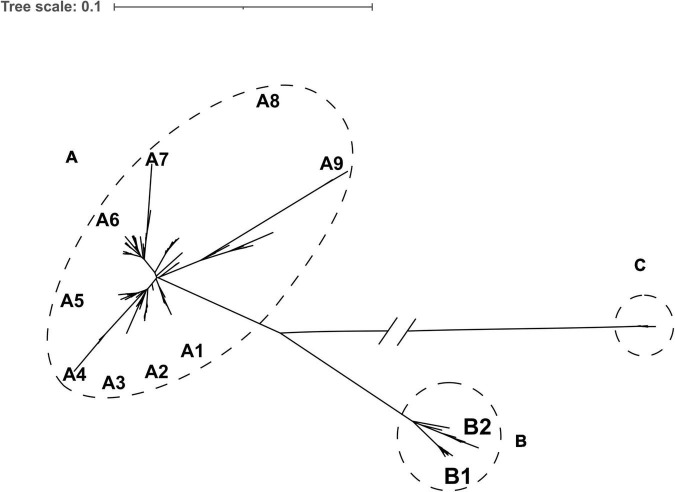
Core genome single nucleotide polymorphism (SNP) phylogeny of 152 KPC-2-producing sequence type 11 (ST11) isolates. Dotted circles and ovals indicate the three major clades (clades A, B, and C). Letters depict subclades described in the text.

**FIGURE 3:**
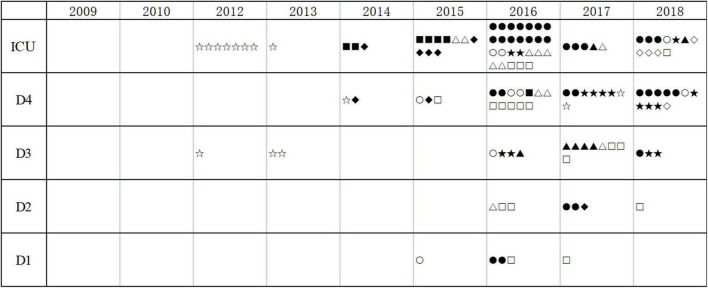
The distribution of KPC-2-producing CRKP ST11. ICU, intensive care unit; D1 to D4, four wards of the respiratory department. Clinical isolates with different types are indicated by various symbols (▲: A1; ▼: A2; ◆: A3; ✱: A4; ◼: A5; ●: A6; ❤: A7; ★: A8; ✿: A9; ○: B1; □: B2; and #: C).

A maximum-likelihood phylogenetic tree was constructed based on core genome single nucleotide polymorphisms (cgSNPs) of the 152 KPC-2-producing CRKP ST11. Three genetically distinct populations, clade A (*n* = 122), clade B (*n* = 22), and clade C (*n* = 8), were observed (Figure 2 and Supplementary Table 2). The isolates of clade A and clade B were further divided into nine and two subclades, respectively. Clade A emerged in 2012 and continued to spread among the different departments until 2018. Clade B emerged in 2016 and spread through 2018. Clade C was associated with an outbreak that occurred between 2014 and 2016 (Figure 3).

### Capsular Polysaccharide Synthesis Gene Analysis

In this study, the majority of clade A belonged to KL47 (*n* = 117) and the rest strains were identified as KL25 (*n* = 5). Isolates of clade B and clade C belonged to KL64 and KL16, respectively (Supplementary Table 2).

### Analysis of *bla*_KPC–2_-Carrying Plasmids

The complete genome sequences of fifteen strains were analyzed. All the *bla*_KPC–2_ genes were located on the plasmids. The characteristics of *bla*_KPC–2_-carrying plasmids are given in Table 1. There were two copies of *bla*_KPC–2_ in tandem on the plasmid in pKPC-IR12079 and pKPC-IR5077.

**TABLE 1:** Characteristics of *bla*_KPC–2_-carrying plasmids.

Strain No.	Strain type	Isolation date	KL type	*bla*_KPC–2_-carrying plasmids			
				Name	Types	Size (bp)	Antibiotic resistance genes
IR12094	A1	01/2017	KL47	pKPC2-IR12094_1	IncFII(pHN7A8)/IncR	130,677	*bla*_KPC–2_, *bla*_CTX–M–65_, *bla*_TEM–1B_, *bla*_SHV–12_, *rmtB*, *fosA3, catA2*
IR12099	A2	01/2017	KL47	pKPC2-IR12099_1	IncFII(pHN7A8)/Inc_pA1763–KPC_	128,678	*bla*_KPC–2_, *bla*_SHV–12_
IR5077	A2	08/2013	KL47	pKPC2-IR5077_4	IncR	92,812	*bla*_KPC–2_ (2 copies)
IR1272	A3	12/2015	KL47	pKPC2-IR1272_1	IncFII(pHN7A8)/Inc_pA1763–KPC_	106,759	*bla*_KPC–2_, *bla*_CTX–M–14b_
IR12243	A4	07/2018	KL25	pKPC2-IR12243_2	IncFII(pHN7A8)/Inc_pA1763–KPC_	147,322	*bla*_KPC–2_, *bla*_CTX–M–65_, *bla*_TEM–1B,_ *bla*_SHV–12,_ *rmtB, fosA3*
IR12182	A5	10/2017	KL47	pKPC2-IR12182_1	IncFII(pHN7A8)/Inc_pA1763–KPC_	131,777	*bla*_KPC–2_, *bla*_TEM–1B_, *rmtB*
IR12079	A6	12/2016	KL47	pKPC2-IR12079_3	IncFII(pHN7A8)	85,555	*bla*_KPC–2_ (2 copies), *bla*_CTX–M–65_, *bla*_TEM–1B_, *rmtB*, *fosA3*
IR12073	A7	11/2016	KL47	pKPC2-IR12073_3	IncFII(pHN7A8)/IncR	153,836	*bla*_KPC–2_, *bla*_CTX–M–65_, *catA2*
IR12024	A8	06/2016	KL47	pKPC2-IR12024_2	IncFII(pHN7A8)/IncR	162,611	*bla*_KPC–2_, *bla*_CTX–M–65_, *bla*_TEM–1B_, *bla*_SHV–12_, *rmtB*, *fosA3*
IR5726	A8	08/2017	KL47	pKPC2-IR5726_3	IncFII(pHN7A8)/IncR	144,355	*bla*_KPC–2_, *bla*_CTX–M–65_, *bla*_SHV–12_
IR1230	A9	02/2013	KL47	pKPC2-IR1230_3	IncFII(pHN7A8)/Inc_pA1763–KPC_	147,295	*bla*_KPC–2_, *aac(3)-IId*, *aph(3″)-Ib*, *aph(6)-Id*, *sul1*, *sul2*, *dfrA25*
IR12183	A9	10/2017	KL47	pKPC2-IR12183_5	IncN	59,999	*bla*_KPC–2_, *aac(3)-IId*, *sul2*, *dfrA1*
IR12197	B1	12/2017	KL64	pKPC2-IR12197_4	IncFII(pHN7A8)/IncR	139,381	*bla*_KPC–2_, *bla*_CTX–M–65_, *rmtB*
IR12061	B2	10/2016	KL64	pKPC2-IR12061_3	IncFII(pHN7A8)/IncR	57,847	*bla*_KPC–2_, *bla*_CTX–M–65_
IR1251	C	04/2015	KL16	pKPC2-IR1251_3	Inc_pA1763–KPC_	86,878	*bla*_KPC–2_, *bla*_TEM–1C_

### Genetic Environment of *bla*_KPC–2_

**FIGURE 4:**
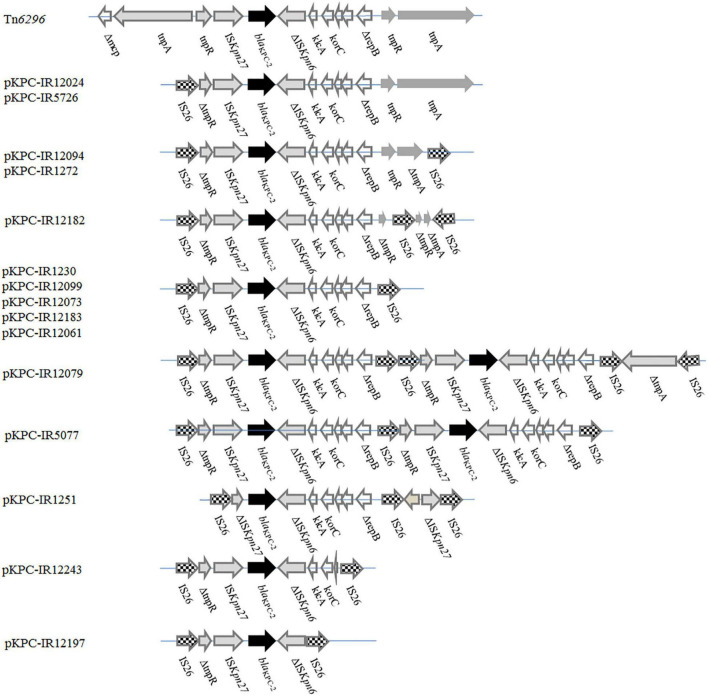
Linear comparison of *bla*_KPC–2_ genetic surroundings between the 15 plasmids. Genes are denoted by arrows. Resistance genes, mobile elements, and other features are shown based on functional classification. Two copies of the IS*26*-ΔtnpR-IS*Kpn27-bla*_KPC–2_-IS*Kpn6*-klcA-korC-ΔrepB-IS*26* units are arranged in tandem in pKPC-IR12079, while in pKPC-IR5077 sharing one IS*26* at the connection.

The genetic environment of *bla*_KPC–2_ showed nine distinct variants compared with the Tn*6296* structure (accession no. FJ628167). All the nine forms were IS*26*-based composite transposons that had a basic core structure (IS*Kpn27-bla*_KPC–2_-ΔIS*Kpn6*). These variants presented different lengths and were truncated by IS*26* at one or both ends of Tn*6296* in different positions. The *bla*_KPC–2_ was commonly present as a single copy on a plasmid, while two copies of *bla*_KPC–2_ were located on a single plasmid of pKPC-IR12079 and pKPC-IR5077, respectively. Meanwhile, two copies of the backbone structure (IS*26*-ΔtnpR-IS*Kpn27-bla*_KPC–2_-ΔIS*Kpn6-klcA-korC*-Δ*repB*-IS*26*) were arranged in tandem with pKPC-IR12079, while they shared one IS*26* at the connection in pKPC-IR5077 (Figure 4).

### Analysis of Virulence Genes-Carrying Plasmids

**FIGURE 5:**
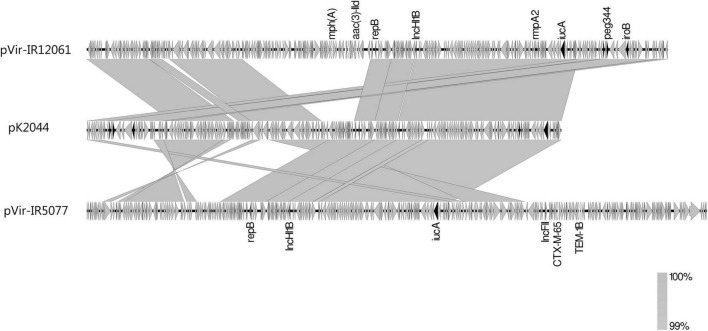
Linear comparison of virulence plasmids pVir-IR12061_1, pVir-IR5077_3, and pK2044. Virulence genes are shown by dark arrows. Other genes are denoted by gray arrows. Regions of synteny between adjacent schematics are indicated by the gray-shaded areas.

About 27.6% (42/152) isolates, including all the subclade A6 isolates (34/34), 6 of 9 subclade B2 isolates, and 2 of 8 subclade A1 isolates, co-carried 2 to 4 virulence marker genes (namely, *peg344*, *iucA*, *iroB*, *rmpA*, and *rmpA2*) for hvKp strains. Three isolates from these subclades: IR12061 (B2), IR5077 (A1), and IR12079 (A6) were analyzed to identify the characteristics of these virulence-carrying plasmids (Table 2). The plasmids pVir-IR12061 and pVir-IR5077 showed high nucleotide sequence identity of more than 99% over the 75 and 65% query coverage with pK2044. Both the plasmids got a resistant fragment based on pK2044 (Figure 5). The pVir-IR12079 from subclade A6 harbored 2 virulence genes and showed 34% query coverage with pK2044.

**TABLE 2:** Characteristics of virulence plasmids.

Strain No.	Strain type	Virulence plasmids				
		Name	Size (bp)	Types	Virulence genes	Resistance genes
IR12061	B2	pVir-IR12061_1	274,502	IncHI1B/repB	*iucA, iroB*, *rmpA*, *rmpA2, peg344*	*aadA5, aac(3)-IId, dfrA17, sul1, mph(A)*
IR5077	A1	pVir-IR5077_3	292,919	IncHI1B/InFII/repB	*iucA*,Δ*rmpA2*	*rmtB*, *bla*_TEM–1B_, *bla*_CTX–M–65_, *fosA3*
IR12079	A6	pVir-IR12079_2	230,866	Non-type	*iucA*,Δ*rmpA2*	*aac(6’)-Ib-cr, sul1, ARR-3, catB3*, *bla*_OXA–1_

## Discussion

In this study, we retrospectively investigated the characteristics of KPC-2-producing CRKP ST11 isolated from the respiratory departments over 9 years. We chose the respiratory departments consisting of four ordinary departments and an ICU, which were one of the most common CRPK outbreaking areas in our hospital. The molecular characteristics of the CRKP in these departments could to some extent elucidate the relevant mechanisms related to the emergence, outbreak, and persistence of CRKP in the whole hospital.

Consistent with other reports in China (Zhang et al., 2017; Dong et al., 2018b), KPC-2-producing CRKP ST11 was first discovered in January 2012 and then quickly became the main epidemic clone (Figure 1). The genetic basis of the predominant prevalence of ST11 in China was not well understood. Some studies revealed that ST11 clone may possess features such as a type IV secretion system in ICEKp258.1 and chromosomal recombination, which may potentially contribute to the dissemination of ST11 (Chin et al., 2018). So, it is important to identify the intrinsic factors that caused the rapid dissemination of ST11 and focus on CRKP infectious control and surveillance. Unlike ST258 associated both with KPC-2 and KPC-3(Marsh et al., 2019), ST11 was strictly associated with KPC-2 production. Further study was needed to understand the mechanism of the association such as the adaptability of plasmids encoding KPC-2 in ST11.

In concordance with previous reports, all the KPC-2-producing ST11 isolates were resistant to multiple antibiotics, namely, aminoglycosides, fluoroquinolones, and cephalosporins (Wang et al., 2016). Fortunately, all the isolates in this study were susceptible to tigecycline and colistin, suggesting a choice for clinical treatment.

Phylogenetic analysis demonstrated that KPC-2-producing CRKP ST11 could be divided into three clades and eleven subclades, which suggested that the strains were highly heterogeneous. The spread of these 12 high-risk clones caused several persistent outbreaks in the whole respiratory departments during the 9 years (Figures 2, 3). A hospital in Beijing analyzed the CRKP outbreak in the ICU within 14 months and found that there were three clones (Li et al., 2020) and analysis of ST11 strains in a hospital in Shanghai found twelve clones from 2009 to 2016 (Liu et al., 2018). Even during an outbreak, there might be one or two clones (Chen et al., 2019). All these results indicated that ST11 KPC-2-producing CRKP evolved separately in distinct clonal groups.

CPS of CRKP is a hotspot of genome recombination and plays an important role in the evolution of bacterial resistance, which is possibly related to the spread and epidemic of CRKP. In this study, basically three noticeably divergent clades were coherent with the results of their KL type. Thus, KL typing may be an important factor for the subgroup of isolates belonging to the same ST type. CRKP KL47 had been widely reported in China in recent years, mainly in the ST11 type (Dong et al., 2018b; Hu et al., 2020). CRKP KL47 is not a highly virulent strain, but in recent years, many reports have found that KL47-type strains can also acquire virulence plasmids and become highly virulent strains with high lethality (Gu et al., 2018). In this study, it was also found that the CRKP KL47-A6 strain carried *rmpA2* and *iucA* genes. In some studies, the CRKP KL64 strain was found to be highly virulent and the isolation rate had gradually increased in recent years (Zhao et al., 2020; Zhou et al., 2020). In this study, although the CRKP KL64 strain did not account for a large proportion, there were also highly virulent strains, which possessed all the virulence genes. It is necessary to continuously monitor the epidemic situation for these clones. Among these clades, we noticed that clade C isolates (KL16) caused a small outbreak in ICU between 2015 and 2016 and then disappeared in the departments. Most of these isolates co-possessed *bla*_KPC–2_ and *bla*_OXA–48_. We analyzed these isolates in another study and found that those plasmids also showed a divergent evolution (Wang et al., 2021).

Compared with the background of *bla*_KPC–2_ gene in Tn*6296*, this study exhibited a considerable diversity of structures surrounding *bla*_KPC–2_ due to the IS*26* insertion; however, they had a similar core structure, IS*Kpn27-bla*_KPC–2_-IS*Kpn6*, as reported in many other studies (Zhang et al., 2017; Dong et al., 2018b). Form IV was found on seven different plasmids, namely, dual replicon IncFII (pHN7A8)/IncR, IncFII (pHN7A8)/Inc_pA1763–KPC_, and IncR plasmids, which belonged to the distinct clones and was identical to Δ*Tn6296-3* in p12181-KPC (Feng et al., 2017). These results suggested that horizontal transfer of Tn*6296* might be the major mechanism responsible for the emergence and rapid transmission of *bla*_KPC–2_. There is no clear relationship between the genetic environment and the plasmid types or the clonal profile of the isolates. After transfer to a plasmid, Tn*6296* evolved continuously. There were two copies of Δ*Tn6296* arranged in tandem in pKPC-IR12079 and pKPC-IR5077, which was similar to the three copies of the IS*26*-IS*Kpn27-bla*_KPC–2_-IS*Kpn6*-IS*26* unit in a study by Feng et al. (2019). We also found that most of the units were truncated by the IS*26* insertion at different sites and there were seven distinct forms ofΔ*Tn6296* (Figure 4). Previous studies also had shown that IS*26* is involved in the dissemination and amplification of *bla*_KPC_ (He et al., 2015) and might generate tandem repeats *via* homologous recombination.

In this study, *bla*_KPC–2_-bearing plasmids were structurally divergent, as various types and sizes were detectable in the strains. Among these, 12 out of 14 plasmids from different subclones harbored IncFII_pHN7A8_ replicon (Table 1). A recent report indicated that the IncFII-like plasmid may promote the spread of the *bla*_KPC_ gene in *K. pneumoniae* ST11 in China (Fu et al., 2019). IncFII plasmids can be divided into multiple subgroups, namely, IncFII_Y_, IncFII_K_, IncFII_pHN7A8_, and IncFII_p0716–KPC_ (Qu et al., 2019). They are usually large (>100 kb), but with low copy numbers and often carry an additional replicon type to initiate replication (Ogbolu et al., 2013). In this study, dual replicons such as IncFII_pHN7A8_ replicon/IncR and IncFII_pHN7A8_/Inc_pA1763–KPC_ had also been reported in other studies in China (Feng et al., 2019; Li et al., 2022). Inc_pA1763–KPC_ replicon often coexists with an auxiliary replicon with the major replicons (such as IncFII and IncR) to compose multireplicon plasmids, represented by plasmids p0716-KPC (Feng et al., 2017). As the sole replicon in the plasmid, the Inc_pA1763–KPC_ replicon is also capable of maintaining the genetic stability of the plasmid (Qu et al., 2019; Chen et al., 2021). In this study, 5 of 14 plasmids harbored dual replicons IncFII_pHN7A8_/Inc_pA1763–KPC_, while one plasmid harbored sole Inc_pA1763–KPC_ replicon. Although some plasmids harbored the same replicon, the *bla*_KPC_-harboring plasmids in different subclades showed different sizes and carried different kinds of resistant genes, suggesting the diversity of the *bla*_KPC_-harboring plasmids.

The hypervirulent KPC-2-producing *K. pneumoniae* ST11 was first identified in 2018 (Gu et al., 2018). The genetic mechanism by which classic ST11 CRKP strains acquired hypervirulence relies on the horizontal acquisition of a pLVPK-like virulence plasmid. In this study, all the hypervirulent isolates obtained a plasmid containing both the virulence genes and resistant genes. Multidrug-resistant (MDR)-virulent plasmids were rare, but an increasing number of studies had been reported in the past few years (Bialek-Davenet et al., 2014; Dong et al., 2018a; Wyres et al., 2020). The emergence and spread of MDR-virulent plasmids as a result of ongoing recombination was a significant potential health threat. Findings in this study provided evidence for active plasmid evolution in *K. pneumoniae* and suggested an urgent need for the surveillance of multidrug-resistant and hypervirulent *K. pneumoniae*.

## Conclusion

In conclusion, using WGS-based analysis, we described a population snapshot of CRKP ST11 collected from the respiratory department between 2010 and 2018 in our hospital. Different clones harbored different plasmids, while diverse genetic environments of *bla*_KPC–2_ gene suggested different sources of acquisition. This finding demonstrated that hypervirulent and multidrug-resistant *K. pneumoniae* strains had already appeared several years ago.

This study has some limitations. First, a collection of about 152 strains across a long period in the respiratory departments may not reflect the whole evolution of the KPC-2-producing ST11 CRKP in a hospital. Second, the complete sequence of one plasmid from each subclade may not reflect the characteristics of all the plasmids from the subclade. Third, the strains are mainly isolated from sputum and urine, which are not easy to check whether these strains cause infection or colonization. Finally, we did not phenotypically evaluate the virulence potential of *K. pneumoniae* clones encoding virulence traits.

## Data Availability Statement

The datasets presented in this study can be found in online repositories. The names of the repository/repositories and accession number(s) can be found below: https://www.ncbi.nlm.nih.gov/genbank/, PRJNA782392; https://www.ncbi.nlm.nih.gov/genbank/, PRJNA835045.

## Author Contributions

DS and JY designed the study. LG, LW, QZ, LY, KY, and YM did the phenotypic and genotypic analyses. LG and JY drafted the manuscript. All authors have read and approved the final version of the manuscript.

## Conflict of Interest

The authors declare that the research was conducted in the absence of any commercial or financial relationships that could be construed as a potential conflict of interest.

## Publisher’s Note

All claims expressed in this article are solely those of the authors and do not necessarily represent those of their affiliated organizations, or those of the publisher, the editors and the reviewers. Any product that may be evaluated in this article, or claim that may be made by its manufacturer, is not guaranteed or endorsed by the publisher.

## Footnotes

1
http://bigsdb.pasteur.fr/klebsiella/

2
http://www.genomicepidemiology.org/services/

3
https://github.com/katholt/Kleborate

4
https://cge.food.dtu.dk/services/CSIPhylogeny/

5
https://itol.embl.de
